# Diagnostic Challenge in Frontal Variant Alzheimer's Disease With Low Amyloid‐β PET Retention

**DOI:** 10.1002/acn3.70025

**Published:** 2025-03-05

**Authors:** Ryosuke Shimasaki, Masanori Kurihara, Kenji Ishibashi, Aya Midori Tokumaru, Kenji Ishii, Atsushi Iwata

**Affiliations:** ^1^ Department of Neurology Tokyo Metropolitan Institute for Geriatrics and Gerontology Tokyo Japan; ^2^ Integrated Research Initiative for Living Well With Dementia Tokyo Metropolitan Institute for Geriatrics and Gerontology Tokyo Japan; ^3^ Research Team for Neuroimaging Tokyo Metropolitan Institute for Geriatrics and Gerontology Tokyo Japan; ^4^ Department of Diagnostic Radiology Tokyo Metropolitan Institute for Geriatrics and Gerontology Tokyo Japan

**Keywords:** amyloid, atypical Alzheimer's disease, biomarker discordance, cerebrospinal fluidbiomarkers, frontal, MK6240, PET, tau PET

## Abstract

Diagnosing frontal variant Alzheimer's disease (fvAD) is difficult and could be even more difficult when amyloid‐beta (Aβ) PET retention is low. A 63‐year‐old woman presenting with a 3‐year history of apathy and memory impairment showed executive dysfunction, memory impairment, and severe bilateral frontotemporal atrophy on MRI. Aβ PET showed only equivocal findings in the right frontal lobe and was negative. However, CSF showed a severely decreased Aβ42/40 ratio and increased phospho‐tau181. AD‐tau‐specific (18F)‐MK6240 PET revealed increased tracer retention predominantly in the bilateral frontal lobes, confirming the fvAD diagnosis. (18F)‐MK6240 PET can be valuable in resolving diagnostic uncertainties in atypical patients with low Aβ retention.

## 1 Introduction

Frontal variant Alzheimer's disease (fvAD) is an atypical presentation of Alzheimer's disease (AD) characterized by prominent behavioral and dysexecutive symptoms [1]. Differentiating fvAD from non‐AD frontotemporal dementia (FTD) based on clinical presentation, brain MRI, or fluorodeoxyglucose (FDG) PET can be challenging [1]. Although amyloid‐β (Aβ) PET has been considered the gold standard for confirming AD pathology, its sensitivity is not absolute, resulting in diagnostic uncertainty due to false negatives in rare cases [2, 3], leading to a diagnostic challenge. Studies have reported on the utility of [18F]‐MK6240, a second‐generation tau tracer with high affinity and selectivity for AD tau (3R/4R tau) in the diagnosis of AD in general [4]. Herein, we report the diagnostic challenges encountered in a case of fvAD with low or equivocal Aβ PET retention, highlighting the clinical utility of [18F]‐MK6240 PET.

## 2 Methods

### 2.1 Patient

A 63‐year‐old woman suspected of having fvAD was enrolled in this study.

### 2.2 Cerebrospinal Fluid Biomarkers

Cerebrospinal fluid (CSF) was obtained via standard lumbar puncture. Concentrations of Aβ40, Aβ42, total tau, and phosphorylated tau 181 (p‐tau181) were measured using the LUMIPULSE system (FUJIREBIO INC., Tokyo, Japan) and its assays [5].

### 2.3 PET Imaging

The patient underwent (18F)‐FDG, (11C)‐Pittsburgh compound B (PiB), and (18F)‐THK5351 PET (visualizing astrogliosis by binding MAO‐B), as previously reported [6, 7]. Due to discordant CSF and amyloid PET results, an additional (18F)‐MK6240 PET scan was conducted. Emission data were acquired 90–120 min following intravenous (18F)‐MK6240 administration. All PET images were visually evaluated by two experts (K. Ishibashi and K. Ishii).

### 2.4 Research Ethics and Patient Consents

This study was approved by the Institutional Review Board of our institution. Written informed consent was obtained from the patient/husband for PET and CSF studies and for this case report.

## 3 Results

A 60‐year‐old, right‐handed woman presented to another hospital due to personality changes noted by her husband, described as a gradual decline in functional abilities (e.g., cooking, cleaning), loss of interest in doing hobbies, and excessive television watching. A brief cognitive assessment at age 61 suggested recent memory impairment; however, a brain MRI demonstrated accentuated atrophy in the bilateral frontal lobes. By age 62, she was unable to maintain her daily life due to an inability to perform household chores. The persistence of symptoms prompted a consult at our clinic by age 63.

**FIGURE 1:**
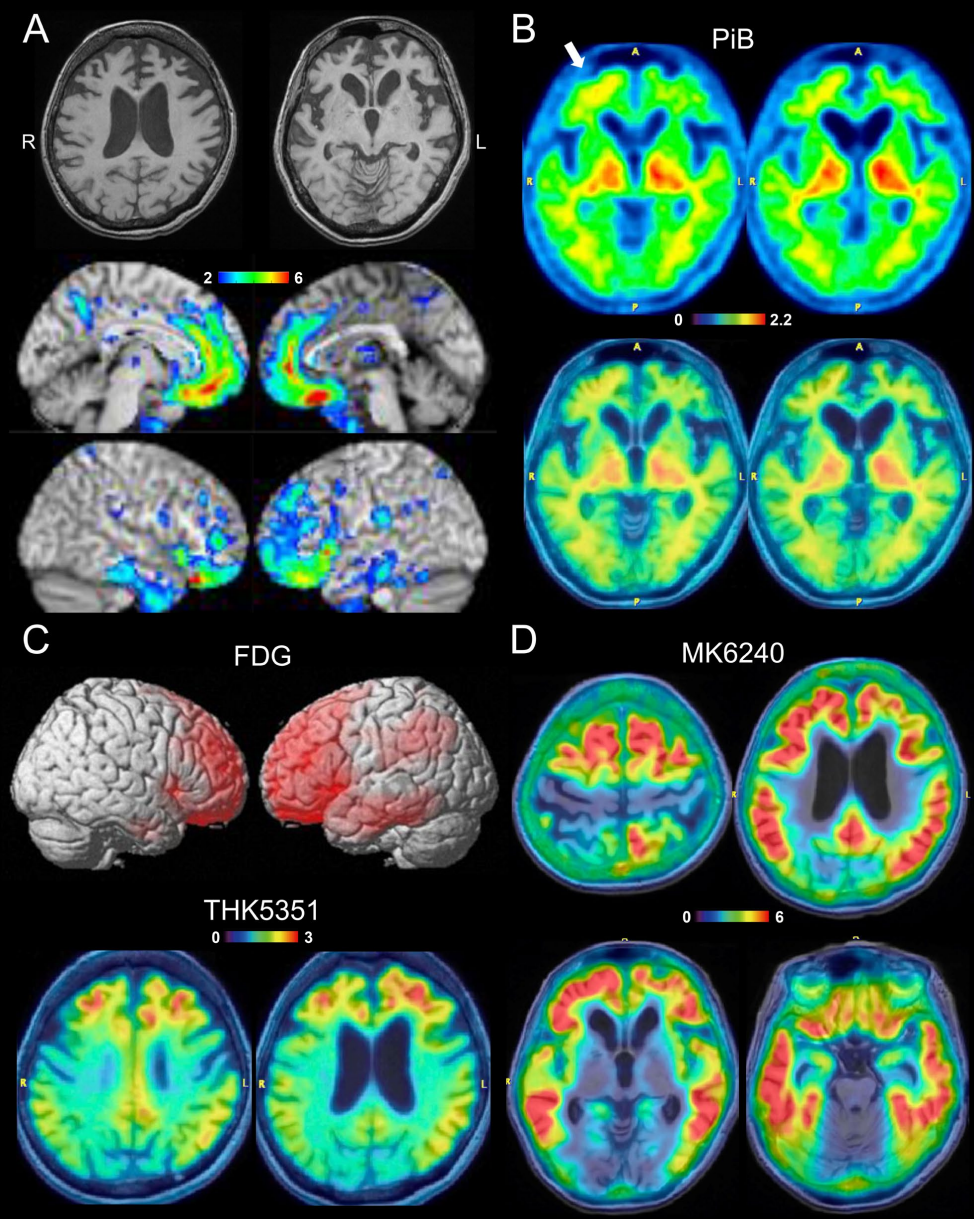
Radiological findings of a patient with frontal variant Alzheimer's disease (AD) with low amyloid‐β PET retention. (A) T_1_‐weighted MRI and volumetric analysis of the gray matter (color scale represents Z‐score). Severe atrophy is mainly observed in the bilateral frontotemporal lobes. (B–D) PET images were rescaled by setting cerebellar uptake to one and are displayed on individual MRI. (B) Aβ PET using (11C)‐Pittsburgh compound B (PiB). Cortical retention is negative in most areas, except for the right frontal lobe showing equivocal findings (arrow). Thus, the binary reading is considered equivocal or negative. Centiloid scale was 5.3, which is also below the widely adopted thresholds. (C) The results of (18F)‐fluorodeoxyglucose (FDG) and (18F)‐THK5351 PET (binds MAO‐B and detects astrogliosis related to neurodegeneration). Statistical parametric mapping (SPM) analysis illustrating decreased brain regions (*p* < 0.05) is shown for FDG PET. While abnormalities in FDG and THK5351 include AD‐related brain areas such as the left lateral temporo‐parietal lobe, posterior cingulate, and precuneus, the most prominent abnormalities are centered in the bilateral frontal lobes. (D) AD‐tau specific (18F)‐MK6240 PET reveals significantly increased tracer retention in the neocortex, including high retention in bilateral frontal lobes, supporting fvAD diagnosis.

She has no family history of dementia or neurological disease. Neurological examination revealed apathy, attention deficit, echolalia, and bilateral grasping reflexes. While her speech was fluent and grammatically unimpaired, her responses were low in volume and often non‐committal (“I don't know”). Her Mini Mental State Examination (MMSE) score was 14/30, with significant deficits in orientation, attention/calculation, and delayed recall. Frontal Assessment Battery (FAB) score was 7/18. Detailed interview with her husband did not disclose behavioral symptoms other than apathy/inertia during the course. Brain MRI showed severe atrophy predominantly in the bilateral frontotemporal lobes (Figure 1A).

Although FTD was suspected, fvAD was on the differential diagnosis. CSF biomarker and PET studies were subsequently performed to confirm the diagnosis. Aβ PET using PiB only showed equivocal cortical retention in the right frontal lobe, which was considered an equivocal or negative binary reading (Figure 1B). While abnormalities on FDG and THK5351 included AD‐related brain areas, abnormalities centered in the bilateral frontal lobes (Figure 1C). Based on these PET findings, non‐AD FTD was suspected. However, CSF assessment indicated AD with a low Aβ42/40 ratio (0.039, cut‐off: 0.067), high p‐tau181 (172.6 pg/mL, cut‐off: 56.5), and high total tau (1236 pg/mL, cut‐off: 404). *APOE* genotype was ε3/ε3.

The discordant results from Aβ PET and CSF biomarkers presented a diagnostic challenge due to the low pre‐test probability of AD based on the atypical frontal presentation. To clarify the diagnosis, an additional PET study using (18F)‐MK6420, a recently developed AD‐tau specific tau tracer, was performed [8]. This imaging modality showed extensive and highly increased tracer retention in the neocortex, including high retention in bilateral frontal lobes (Figure 1D), confirming the diagnosis of fvAD.

At the time of diagnosis, the patient was ineligible for disease‐modifying treatment. Symptomatic management with an acetylcholinesterase inhibitor was initiated, significantly improving the patient's apathy and increasing engagement in communication.

## 4 Discussion

We presented a diagnostically difficult case of fvAD with low or equivocal Aβ PET retention. Discordant results in CSF AD biomarkers led to further evaluation, and additional (18F)‐MK6240 PET supported the diagnosis of fvAD.

Previous studies have shown that fvAD may represent more than 10% of patients with clinically diagnosed FTD [9]. AD biomarkers are crucial for early diagnosis and have become more important in the current era of available disease‐modifying therapies. In this case, although the patient presented to our clinic at an advanced stage, earlier biomarker testing and diagnosis may have led to a more favorable prognosis.

While Aβ biomarker results are generally concordant between CSF and PET, discordant results have been reported in approximately 10%–20% of cases [10, 11, 12]. One hypothesis suggests that biomarker discordance represents a transitional phase in the progression of Aβ pathology, where individuals transition from a normal CSF/PET to a discordant state (CSF+/PET− or CSF−/PET+) and eventually present with CSF+/PET+ [11]. However, this hypothesis primarily assumes that biomarker discordance occurs in the preclinical or prodromal phase and is considered rare in advanced stages [10, 11, 12]. Additionally, most previous studies utilized CSF Aβ42 alone, which is less accurate compared to the recent Aβ42/40 ratio. Moreover, diagnoses in these studies relied on clinical judgment alone, and the distribution of tau PET abnormalities or CSF p‐tau181 values, which may be associated with the distribution of tau pathology [13], was not described in detail. Therefore, it has remained unclear whether patients with advanced AD and high neocortical tau PET uptake could also exhibit discordant Aβ biomarker results. Our case demonstrated that Aβ biomarker discordance can be observed in relatively advanced AD with very high CSF p‐tau and neocortical tau uptake, even when using the accurate Aβ42/40 ratio.

Previously, low or negative Aβ PET retention in symptomatic AD was considered a rare phenomenon observed only in rare forms of autosomal‐dominant AD. However, recent studies have reported on low Aβ PET and high neocortical tau in non‐familial cases, extending beyond the regions typically seen in primary age‐related tauopathy [8, 14]. Although CSF biomarker results were available only in some of these cases, the findings supported the diagnosis of AD, and authors suggested that Aβ PET may not have detected the Aβ plaques present in these cases [14]. These low amyloid‐beta and high neocortical tau PET cases have been reported in 1.4% of Aβ PET negative individuals in a recent study [14], and may be higher in symptomatic patients. Our findings are consistent with these recent reports and further validated using approved high‐accuracy CSF biomarkers.

Several hypotheses have been proposed for the mechanism of Aβ biomarker discordance. One hypothesis attributes the discordance to the fact that CSF and PET reflect different aspects of Aβ aggregation in the brain. While CSF Aβ42 levels are considered to reflect the rate of Aβ aggregation, including non‐fibrillar forms, Aβ PET measures the density of fibrillar Aβ deposition. In a report by Cairns et al., an 89‐year‐old male with negative PiB PET and abnormal CSF AD biomarkers was found on neuropathological examination to have diffuse amyloid plaques but only sparse neuritic plaques, suggesting that the relative absence of fibrillar Aβ plaques likely contributed to the negative PiB PET finding [2]. Another hypothesis posits that unusual Aβ deposition, such as cotton wool plaques known to weakly bind Aβ PET tracers, may be involved. However, this has not been confirmed in non‐genetic AD. The third hypothesis indicates that Aβ PET false negativity may be associated with cortical atrophy [15]. In our patient, the equivocal Aβ PET findings observed in the right frontal lobe may have been influenced by significant frontal cortical atrophy.

Recently, there have been significant advancements in blood‐based biomarkers for AD. Since autopsy validation can be difficult to conduct in a large sample size, most studies also use Aβ PET as the gold standard. However, if only Aβ PET was conducted, discordant cases like this case would be labeled as non‐AD control, which could affect the diagnostic performance in the study. Moreover, blood and CSF biomarkers do not necessarily correlate tightly [16] and could also be discordant [17, 18]. Therefore, comprehensive studies including blood, CSF, amyloid PET, and tau PET could be important to better understand the true potential of blood‐based biomarkers for AD.

There are several limitations to this study. First, this was a single case report, and replication in a larger study is necessary. Second, the diagnosis of fvAD is difficult to fully prove antemortem, and confirmation in neuropathological analyses at autopsy is important, especially to rule out the presence of concomitant pathologies other than AD. Third, although autoradiographic studies have shown that (18F)‐MK6240 does not bind to TDP‐43 or tau aggregates other than AD‐type neurofibrillary tangles [4], and clinical studies so far have not reported false positive results other than low tracer retention in *MAPT* mutation carriers (R406W and P301L) [19] that are distinguishable from high retention in AD, autopsy‐validation studies are needed to further confirm the specificity of in vivo (18F)‐MK6240 PET.

In conclusion, fvAD with low amyloid PET retention can pose a diagnostic challenge. The presence of discordantly abnormal CSF AD biomarkers could be suggestive of this condition, warranting further evaluation with PET using AD‐tau specific tracers, such as (18F)‐MK6240, to confirm its diagnosis in complex cases.

## Author Contributions

The author takes full responsibility for this article.

## Conflicts of Interest

Masanori Kurihara has received a patent assignment fee from FUJIREBIO INC. Atsushi Iwata has received research grants, a patent assignment fee, and advisory/speaker fees from FUJIREBIO INC. Kenji Ishii has received research support from Lantheus/Cerveau Technologies and Nihon Medi‐Physics, and consultancy/speaker fees from Nihon Medi‐Physics. Ryosuke Shimasaki, Kenji Ishibashi, and Aya Midori Tokumaru report no disclosures relevant to the manuscript.

## Data Availability

The data supporting the findings of this study are available from the corresponding author upon reasonable request.
